# Knowledge and Awareness of Ionizing Radiation Harms Among Hospital Employees at a Large Tertiary Medical Center: Findings from a First-of-Its-Kind Study in Israel

**DOI:** 10.3390/healthcare13080958

**Published:** 2025-04-21

**Authors:** Ghassan Makhoul, Saritte Perlman, Tomer Ziv-Baran, Gil Fire

**Affiliations:** 1 Department of Radiology, Tel Aviv Sourasky Medical Center, Tel Aviv 6423906, Israel; ghassanmak93@gmail.com; 2 School of Public Health, Faculty of Medical & Health Sciences, Tel Aviv University, Tel Aviv 6997801, Israel; 3 Hospital Administration, Tel Aviv Sourasky Medical Center, Tel Aviv 6423906, Israel; gilf@tlvmc.gov.il; 4 Coller Faculty of Management, Tel Aviv University, Tel Aviv 6997801, Israel

**Keywords:** radiation exposure, radiological safety knowledge, medical radiation awareness, cross-sectional, hospital staff

## Abstract

**Background**: Medical imaging and therapeutic tools are used on a daily basis. Some of these technologies bear potential risk of harm due to exposure to ionizing radiation. Previous research has shown a lack of knowledge and awareness surrounding ionizing radiation among a wide range of medical staff. **Objectives**: This study aimed to evaluate the knowledge and awareness of ionizing radiation in a large tertiary medical center and compare the knowledge and awareness among types of hospital employees. **Methods**: A cross-sectional study based on an anonymous 32-question questionnaire was conducted. Participants were categorized by employee type, including physicians, nurses, allied health professionals, and ancillary and administrative staff. The questionnaire was divided into demographic profile, professional characteristics related to ionizing radiation, and knowledge and awareness. Knowledge and awareness scores were analyzed as standard scores (Z-scores). Univariate and multivariable analyses were performed. **Results**: The study included 479 participants. Physicians received the highest scores compared to other employee types, participants who had received ionizing radiation training received higher scores, and similar trends were observed for employees working in departments with higher potential for radiation exposure. **Conclusions**: This research underscores the need to enhance ionizing radiation knowledge and awareness among hospital staff. Achieving this may involve training sessions, workshops, and academic courses.

## 1. Introduction

Advances in medical technologies over the past decades have given rise to a range of medical imaging and therapeutic tools that enhance and improve the lives of millions of patients on a daily basis. Technologies such as CT scanners, X-ray machines, and fluoroscopy units, as well as more advanced modalities like digital tomosynthesis and positron emission tomography (PET), involve exposure to ionizing radiation for patients and hospital staff [1]. Studies show that exposure to ionizing radiation is associated with an increased risk of malignancy and other illnesses, prompting significant concern [2,3,4,5,6,7,8]. It is estimated that approximately 700 new cancer cases are diagnosed in the UK each year as a result of medical imaging [9].

In the hospital setting, the extent of exposure and the requisite knowledge and awareness significantly vary among different staff roles. Physicians, often directly involved in the application and oversight of radiological procedures, undergo specialized training in operating radiation machines and handling radioactive materials. These employees, known as “radiation workers”, must possess a higher level of knowledge about the risks of ionizing radiation compared to other medical staff in the hospital, with mandatory training sessions conducted semi-annually to ensure up-to-date safety practices [10]. Other hospital employees may work in proximity to radiation sources and are at risk of indirect exposure during their routine duties. These employees may belong to nursing departments, housekeeping, maintenance, security, reception, and clerical staff. Additionally, patient transport teams, operating room staff, and recovery room staff may come into contact with patients undergoing brachytherapy (radioactive implants) and those treated in nuclear medicine. Therefore, it is crucial for physicians to consider the advantages and disadvantages of exposing patients to radiation when recommending and implementing treatment plans, while other hospital employees may encounter direct and indirect exposure to ionizing radiation as part of their daily work activities. However, despite the critical role of training and awareness, studies globally have highlighted the lack of knowledge and awareness of ionizing radiation among a wide range of medical staff, extending from physicians to non-imaging personnel [11,12,13]. Additional studies have shown that physicians have an insufficient level of knowledge and understanding of radiation safety, leading to unnecessary radiation exposure through redundant tests and improper safety practices, underscoring an urgent need for targeted educational interventions [10,14,15,16].

This study aims to bridge a significant gap in the literature by evaluating the levels of knowledge and awareness of ionizing radiation among hospital staff in Israel, a context yet to be thoroughly explored. Despite extensive international research on this topic, there has been no comprehensive assessment among Israeli hospital employees. This study addresses this critical void by examining the diverse roles within a large tertiary medical center known for its substantial use of advanced imaging technologies.

The selected medical center serves a significant portion of the region’s population and stands as a primary teaching hospital, positioning it as a crucial site for potentially influencing national standards and practices regarding radiation safety. This pivotal role makes the medical center an ideal candidate for a study that could inform future radiation education and safety protocols across the country.

The hypothesis of this study posits that significant disparities exist in the levels of knowledge and awareness regarding ionizing radiation safety across different categories of hospital staff. These disparities may influence the adherence to safety protocols and increase the risk of radiation exposure incidents, highlighting the critical need for tailored educational interventions.

## 2. Methods

### 2.1. Study Design, Population, and Setting

A questionnaire-based cross-sectional study at Tel-Aviv Sourasky Medical Center (TASMC), the second largest hospital in Israel, employing over 9000 staff members across various medical and administrative roles.

Employees were divided into four categories: (a) physicians, (b) nursing staff, (c) allied health professionals (i.e., physiotherapists, medical technologists, and nutritionists), and (d) ancillary and administrative staff (porters, orderlies, and medical secretaries). Employees from imaging and radiotherapy departments were excluded from the study to focus on assessing the general awareness and knowledge of ionizing radiation among hospital staff who typically have less direct and frequent exposure to radiation compared to their counterparts in specialized radiological roles. Excluding these departments helped prevent an overestimation of the general awareness level across the hospital, as staff in these departments are expected to have higher knowledge and training in radiation safety due to the nature of their work.

Departments were classified as departments with high or low ionizing radiation use based on the researchers’ professional experiences and expertise. The list of departments and distribution of participants can be found in Appendix A.

Participants were randomly selected from each of the employee categories throughout during various work shifts across all days of the week.

### 2.2. Research Tools, Data Collection, and Study Variables

Knowledge and awareness were evaluated using a 32-question multiple-choice Hebrew-language questionnaire divided into 3 sections (see Supplementary Materials). The first section included demographics such as age, gender, job, level of seniority, and department in which the participant worked. The second section examined participants’ professional characteristics regarding ionizing radiation-related tasks, such as referral of patients for imaging tests, accompanying and informing patients about the tests, having undergone training on ionizing radiation and its potential for harm, and self-assessment of knowledge and awareness. The third section assessed participants’ knowledge about ionizing radiation and behaviors in a radiation environment. Responses to knowledge and awareness questions formed the basis for calculating scores.

The knowledge and awareness section was further divided into 3 levels of difficulty: (1) low-difficulty, basic questions, (2) moderate-difficulty questions requiring broader knowledge, and (3) high-difficulty, complex questions. Level of difficulty was determined by two researchers who are senior physicians with more than 30 years of experience each and was confirmed after consulting with radiographers. Since the questionnaire is not a validated test for assessing knowledge and awareness, and to enable better interpretation of the study results, the scores of the questionnaires were converted to standard scores, which were used for statistical analysis and comparison among the employee populations. The standard score of the total questionnaire for each participant was calculated as the participant score minus the mean score divided by the standard deviation [(participant score − mean score)/standard deviation].

### 2.3. Statistical Methods

Categorical variables were described as numbers and percentages. Continuous variable distributions were evaluated using histograms and Q-Q plots, and they were reported using mean and standard deviation (SD) or median and interquartile range (IQR).

The comparison of the continuous variables that were not normally distributed (age and seniority) and the ordinal variable (self-assessment of knowledge and awareness) among employee categories was analyzed using the Kruskal–Wallis test. Questionnaire standard scores were compared among employee categories using the one-way analysis of variance (ANOVA). The independent samples t-test was applied to compare the questionnaire standard scores between those who received training and those who did not and between those with high and low potential exposure to ionizing radiation. Categorical variables were compared among employee categories using the Chi-square test. Multivariable analysis was performed in order to study the associations among questionnaire standard scores and employee categories while controlling for potential confounders (age, sex, seniority, exposure, and past training). The same method was used to study the association between questionnaire standard scores and past training, as well as potential exposure. Adjusted (adj.) coefficients and 95 percent confidence interval (95% CI) were reported. The linear regression model was used for all multivariable analyses (Standard score = B_Nurses_ × Nurses + B_Allied health professionals_ × Allied health professionals + B_Ancillary and administrative staff_ × Ancillary and administrative staff + B_Age_ × Age + B_Sex_ × Sex + B_Seniority_ × Seniority + B_Potential exposure_ × Potential exposure + B_Past training_ × Past training). All statistical tests were two-sided, *p* < 0.05 was considered statistically significant, and SPSS software was used (IBM SPSS Statistics, version 28, IBM corp., Armonk, NY, USA, 2021).

### 2.4. Sample Size

The sample size calculation was conducted using a significance level of 5% and a power of 80%. In the study, we sought to identify at least a medium effect (effect size f = 0.25) of the employee category on the level of knowledge and awareness of ionizing radiation. Under these assumptions, a total of 180 employees were required. In order to have statistical power to identify a smaller effect, we recruited at least 100 in each group.

### 2.5. Ethical Considerations

The study was approved by the Helsinki Committee at Tel Aviv Sourasky Medical Center and the Ethical Committee of Tel Aviv University (Approval Number: TLV-0353-19). Information, including the researchers’ names, study aims, and statements of confidentiality and complete anonymity, were included on the cover page of the questionnaire. Responding to the questionnaire constituted informed consent to participate in the study. The questionnaire was anonymous and was distributed and returned in a sealed envelope without identifying details.

## 3. Results

### 3.1. Participants Demographics

A total of 479 participants responded to the questionnaire (79.8% response rate); 247 were female (52%), and the median age was 37 years. Demographic characteristics are summarized in Table 1.

### 3.2. Participant Professional Characteristics

Approximately one-third (29.9%) of participants reported that they had received training about the use and potential harms of ionizing radiation. The highest percentage reported was among physicians, 48.8% of whom stated they had undergone ionizing radiation training, while only 1.5% of ancillary and administrative staff reported having received training.

Most participants, 90.4%, believed that training for employees is necessary to explain the potential harms of ionizing radiation. No significant difference was found among employee categories (*p* = 0.095). Overall, participants rated their level of knowledge and awareness with a median score of 4 out of 10 (IQR 3–6). Physicians rated their level of knowledge and awareness with a median score of 5 out of 10, whereas nurses, allied health professionals, and ancillary and administrative staff scored themselves 4 out of 10. Among the study participants, 29.9% referred patients for imaging tests, 39.2% accompanied patients for imaging tests, and 71.2% were present in departments where a mobile X-ray machine was used. Only 37.1% of participants explained to patients or their companions about the tests and the potential harm that may be caused as a result.

Table 2 presents a comparison of the professional characteristics of ionizing radiation-related tasks among the employee categories.

### 3.3. Knowledge and Awareness of Ionizing Radiation Among Hospital Employees

**Figure 1:**
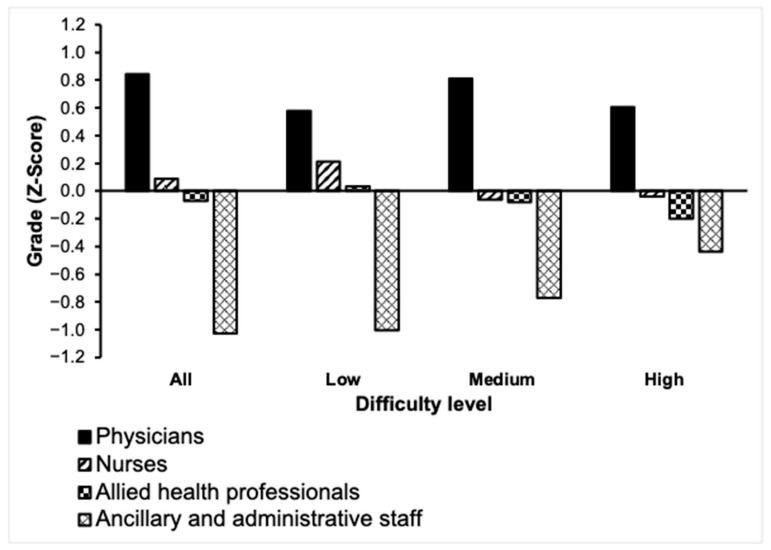
Mean knowledge and awareness scores according to employee type.

Physicians had the highest scores at all levels of difficulty (*p* < 0.001). Significant differences were found among employee categories before and after the stratification of questions by difficulty. Mean scores by employee category are summarized in Table 3 and Figure 1.

Employees were asked to classify whether different imaging methods utilize ionizing radiation; 7% and 22.5% of participants stated that US and MRI, respectively, involve ionizing radiation, while 26.3% and 10.4% claimed that PET and CT scans do not involve exposure to ionizing radiation (Table 4).

When asked to assess the amount of radiation obtained from head, abdominal, chest, and pelvic CT scans, 62% of all participants selected “do not know”. Only 30% of physicians correctly estimated the amount of radiation in a head CT, 12% in an abdominal CT, 21% in a chest CT, 8% in a pelvic CT, and 18% in a mammogram.

In multivariable analysis, after adjustment for age, sex, level of seniority, past training, and potential for exposure, physicians received significantly higher scores compared to nursing staff (mean −0.75, 95% CI −0.94–−0.56, *p* < 0.001), allied health professionals (mean −0.83, 95% CI −1.04–−0.62, *p* < 0.001), and ancillary and administrative staff (mean −1.84, 95% CI −2.07–−1.62, *p* < 0.001). Table 3 summarizes the differences among employees’ overall scores by difficulty level for low, moderate, and high-difficulty questions.

### 3.4. The Association Between Training and Knowledge and Awareness Score

Overall, participants who received training had significantly higher total scores, as well as in each level of difficulty (*p* < 0.01 for all comparisons of the mean scores, Table 5). However, after stratifying by employee type, no significant difference was observed in the total score between employees who received training and those who did not. In the level of difficulty score analysis, physicians who received training had higher scores in the sub-score of low-difficulty questions; similarly, allied healthcare professionals and ancillary and administrative staff who received training had higher scores in the moderate-difficulty questions. A comparison of scores among employees who received training and those who did not is presented in Appendix B.

After adjustment for age, gender, level of seniority, potential for exposure, and employee type, participants who underwent training tended to have a higher total score (*p* = 0.066). However, moderate difficulty sub-score was higher among those who underwent training (mean 0.28, 95% CI 0.11–0.46, *p* = 0.002).

### 3.5. The Association Between the Potential for Exposure and Knowledge and Awareness Score

Overall, participants who worked in high potential exposure departments had significantly higher total scores and sub-scores for level of difficulty (*p* < 0.02 for all comparisons of the mean scores, Table 5). After stratifying by employee type, a significant difference in total score was only observed among nursing staff and allied health professionals. A comparison of scores among employees who worked in high potential exposure departments and those who did not is presented in Appendix C.

After adjustment for age, gender, level of seniority, previous training, and employee type, participants who worked in high potential exposure departments received a higher total score (0.16, 95% CI 0.01–0.31, *p* = 0.036) and a higher moderate difficulty sub-score (0.20, 95% CI 0.04–0.36, *p* = 0.015).

## 4. Discussion

This study assessed the level of knowledge and awareness of ionizing radiation among hospital employees at a large tertiary medical center. The findings demonstrate a lack of knowledge and awareness among the various types of hospital employees and areas of practice. Demographic characteristics were not found to be related to broad and in-depth knowledge of the subject.

All hospital employees, especially clinical staff, such as physicians and nurses, are expected to have knowledge and awareness about ionizing radiation and its potential harms, including whether the imaging method uses ionizing radiation, the level of ionizing radiation, and its short- and long-term effects. The consequences of a lack of knowledge can affect the selection of the ideal imaging methods, leading to unnecessary radiation exposure for both staff and patients. The present study underscores this concern, as a significant proportion of employees reported insufficient knowledge about radiation exposure and protection.

Moreover, data from this present study highlight the lack of awareness about ionizing radiation and suggests hospital employees are exposing themselves and their patients to radiation that may harm their health. A previous study conducted in Israel showed that 74% of nurses and 62% of physicians demonstrated poor knowledge in assessing the levels of radiation that patients are exposed to during CT scans, which aligned with the low-to-moderate knowledge levels observed in this study. Additionally, employees in the imaging departments had an advantage over their colleagues working in the other departments. Overall, 70% of participants reported that their level of knowledge and awareness about the harms of ionizing radiation was low to moderate and they felt the need to strengthen their knowledge [17]. This is consistent with research that healthcare workers often report feeling inadequately prepared to manage radiation risks despite being involved in radiological practices. These previous findings align with the present study, where staff from departments with frequent radiation exposure demonstrated greater knowledge, likely due to their direct engagement with radiological procedures.

A study in Saudi Arabia revealed that only 44% of healthcare workers in radiology departments demonstrated high awareness of radiation risks, further emphasizing the global need for improved radiation safety education [18]. This trend was mirrored by other international studies, where healthcare workers, particularly in non-radiology departments, often struggled to identify basic radiation safety principles [19,20]. Studies have also shown that nurses in various settings, such as intensive care units and operating theaters, often lack sufficient training and knowledge about radiation safety, which impacts their ability to adhere to safety protocols [21,22]. Previous research from Australia assessing nursing staff’s knowledge of radiation protection and practice included 147 nurses from 9 different departments and found a significant difference in the average number of correct responses across different departments. Nursing staff in departments with a high potential for exposure to ionizing radiation received the highest average scores [23]. Similarly, some of the participants in this present study who work in departments defined as having a high potential for exposure to ionizing radiation received higher scores relative to participants from departments where potential exposure to radiation was low.

Overall, one-third of the study participants previously underwent specific training on ionizing radiation and its harms, and almost all study participants (90%) considered it important for employees to receive specific training on the potential harms of ionizing radiation, independent of their previous professional education and training. The data may indicate that staff feel insecure about their levels of knowledge and awareness, which is reflected in their low self-assessment scores. Participants’ high awareness that others need guidance indicates their perception of the issue’s importance.

Multivariable analysis showed that participants who reported having received training in the past tended to have a higher total score (*p* = 0.066) and significantly higher mid-level score (*p* = 0.002). These findings signal the impact and importance of providing training to all employees at the medical center in order to reach and maintain a high level of knowledge and awareness of the potential harms of ionizing radiation. The effectiveness of such training and educational initiatives regarding ionizing radiation is demonstrated by previous research in which 154 cardiologists attended a minicourse on ionizing radiation protection and demonstrated a significant decrease in fluoroscopy times (20.8%) and amount of ionizing radiation absorbed by the body (48.4%) [24]. The impact of previous training is evident in this study as well, with participants who reported prior exposure to radiation safety training tending to score higher on assessments of knowledge and awareness. This aligns with previous findings, where healthcare workers who had received targeted radiation protection training demonstrated better knowledge of safety practices [25,26].

Despite these findings, knowledge gaps remain, particularly regarding more complex aspects of radiation safety, such as the ALARA (as low as reasonably achievable) principle and permissible dose limits [27,28]. Studies indicate that even when healthcare workers demonstrate a basic understanding of radiation protection procedures, deeper knowledge regarding advanced principles remains lacking [25].

Given the significant gaps in knowledge observed across all staff categories, it is essential to implement a multi-tiered educational approach. Targeted training should be integrated into the continuing education of all healthcare workers, with special emphasis on non-radiology departments where awareness is notably lower [18,29]. As demonstrated by other studies, regular in-service training programs tailored to the specific needs of different healthcare roles and introducing more specialized training in the form of radiology seminars or workshops have been shown to enhance awareness and are vital for improving radiation safety knowledge [18,20,30,31]. Moreover, leveraging advanced technologies and digital health tools to monitor and manage radiation exposure can further improve safety practices [32]. These strategies, combined with institutional support and policy changes, can significantly enhance radiation safety awareness and practices among healthcare staff, ultimately improving patient and staff safety in medical centers.

One of the strengths of this study is its inclusion of both clinical and ancillary and administrative hospital employees with both self-assessment and formal assessment of knowledge and awareness of the potential harms of ionizing radiation. Due to the robust study methods and large sample size, research findings can be generalized to other large tertiary medical centers.

Despite its strengths, this study has some limitations. First, the use of multiple-choice questions may lead to biases towards the correct answer. However, the low scores across all participants suggest that this limitation had minimal impact on the findings. Second, to ensure a high response rate, the questionnaire was designed with a limited scope; however, it remained comprehensive enough to capture essential themes. Third, the lack of a validated questionnaire meant that the survey was developed by researchers based on their expertise in radiology and medical management. While rigorous efforts were made to ensure accuracy, using a previously validated tool could have enhanced reliability. Moreover, the questionnaire was analyzed using standard scores rather than absolute values, which allowed for uniform comparisons. However, a weighted or integrated scoring system might have provided a more detailed assessment, particularly by emphasizing critical radiation safety concepts. Fourth, the anonymous nature of the questionnaire prevented response verification, though anonymity was prioritized to reduce reporting bias and encourage honest answers. However, this limitation may introduce recall bias, as participants rely on memory for their responses, and social desirability bias, where individuals may overestimate their knowledge or adherence to safety practices. Fifth, although comparable with other studies, there is no reference score for comparison. Therefore, it is not possible to objectively determine whether the scores obtained are considered satisfactory.

To minimize selection bias, questionnaires were randomly distributed across 22 different departments, covering all work shifts throughout the week. To ensure randomness, surveys were allocated proportionally across departments and shifts and distributed at varied times to capture a diverse mix of staff with different roles and experience levels. Further research should include conducting similar studies in different medical settings and exploring knowledge and awareness among other healthcare settings, including community hospitals and private institutions, to assess whether these trends are consistent across different healthcare environments. Further research should also investigate knowledge levels among medical students, allied health professionals, and even patients and their families, as public awareness of radiation exposure remains an important area of concern. Additionally, longitudinal studies evaluating the impact of structured training programs would be valuable in determining the most effective educational interventions for improving radiation safety knowledge.

## 5. Conclusions

Knowledge and awareness of ionizing radiation vary across different hospital employees, depending on their professional roles, education, and prior training. The present findings suggest that while awareness of radiation hazards exists, knowledge levels remain insufficient, particularly among non-clinical staff and allied health professionals. The absence of strong relationships among demographic characteristics and comprehensive knowledge suggests that simply acquiring more experience or age does not necessarily lead to a better understanding of radiation hazards. This reinforces the need for focused educational initiatives, irrespective of employees’ experience levels.

A substantial number of participants demonstrated gaps in fundamental radiation safety concepts, with lower scores observed as question complexity increased. While physicians obtained the highest scores overall, significant knowledge gaps were found, especially in estimating radiation doses from CT scans. Employees with prior radiation safety training or frequent exposure to imaging procedures tended to have higher knowledge levels. Additionally, participants who regularly accompanied patients to imaging procedures and provided explanations about the exams scored higher, suggesting that direct engagement with radiological practices enhances understanding.

These findings highlight the need for improved education and structured training programs on radiation safety for all hospital employees, especially those outside radiology-intensive departments. Strengthening radiation awareness through targeted interventions could help improve knowledge, promote safer practices, and reduce unnecessary radiation exposure for both staff and patients.

## Figures and Tables

**Table 1:** Participant demographics.

	n = 479
Age (years), median (IQR)	37 (30–45)
Female, n (%)	247 (51.6%)
Seniority (years), median (IQR)	7 (3–16)
Employee type, n (%)	
Physicians	121 (25.3%)
Nurses	150 (31.3%)
Allied health professionals	103 (21.5%)
Ancillary and administrative staff	105 (21.9%)
Departments, n (%)	
Internal medicine	141 (29.4%)
Surgical	164 (34.2%)
Pediatrics	45 (9.4%)
General emergency	25 (5.2%)
Rehabilitation	41 (8.6%)
Porter services	34 (7.1%)
Nutritional services	29 (6.1%)

**Table 2:** Comparison of the professional characteristics of ionizing radiation-related tasks among employee categories.

	All Participants	Physicians	Nurses	Allied Health Professionals	Ancillary and Administrative Staff	*p* ^#^
n	479	121	150	103	105	
Received training in the past	29.9%	48.8%	32.0%	24.3%	1.5%	<0.001
Believe there is a need for a training	90.4%	87.6%	95.3%	89.3%	87.6%	0.095
Self-assessment of knowledge and awareness (scale 1–10); Mean (SD); Median (IQR)	4.3 (2.2); 4 (3–6)	5.2 (2.1); 5 (4–7)	4.3 (2.1); 4 (3–6)	3.8 (2.1); 3 (2–6)	3.6 (2.3); 4 (1–5)	<0.001
Refer patients to imaging tests	29.9%	88.4%	24.0%	N/A	N/A	<0.001
Accompany patients to imaging tests	39.2%	70.2%	33.3%	20.4%	30.5%	<0.001
Explain imaging tests to patients	37.1%	46.3%	20.0%	N/A	N/A	<0.001
Are present in the department during imaging tests	71.2%	79.3%	86.0%	53.4%	60.0%	<0.001
High potential exposure to ionizing radiation	51.6%	59.5%	67.3%	32.0%	39.0%	<0.001

^#^ *p*-values were calculated using the chi-square test or the Kruskal–Wallis test.

**Table 3:** Differences among employees’ scores by employee type and question level of difficulty.

Question Level of Difficulty	Employee Type	Standard Score Mean (SD)	Adj. Coefficient (95% CI) *	*p* ^#^
Overall	Physicians	0.84 (0.75)	Ref.	
	Nurses	0.09 (0.73)	−0.75 (−0.94–−0.56)	<0.001
	Allied health professionals	−0.07 (0.72)	−0.83 (−1.04–−0.62)	<0.001
	Ancillary and administrative staff	−1.03 (0.88)	−1.84 (−2.07–−1.62)	<0.001
Low	Physicians	0.58 (0.65)	Ref.	
	Nurses	0.21 (0.82)	−0.44 (−0.65–−0.23)	<0.001
	Allied health professionals	0.03 (0.85)	−0.56 (−0.78–−0.33)	<0.001
	Ancillary and administrative staff	−1.00 (0.99)	−1.68 (−1.92–−1.44)	<0.001
Moderate	Physicians	0.81 (0.78)	Ref.	
	Nurses	−0.06 (0.80)	−0.75 (−0.96–−0.54)	<0.001
	Allied health professionals	−0.08 (0.90)	−0.68 (−0.91–−0.46)	<0.001
	Ancillary and administrative staff	−0.77 (0.89)	−1.41 (−1.65–−1.17)	<0.001
High	Physicians	0.60 (1.23)	Ref.	
	Nurses	−0.04 (0.90)	−0.65 (−0.89–−0.41)	<0.001
	Allied health professionals	−0.20 (0.77)	−0.77 (−1.03–−0.51)	<0.001
	Ancillary and administrative staff	−0.44 (0.66)	−1.01 (−1.29–−0.74)	<0.001

* Adjusted to age, sex, seniority, exposure, and past training; ^#^ *p*-values were calculated using linear regression.

**Table 4:** False classification rates of ionizing radiation according to imaging method.

Imaging Method	Total Participants	Physicians	Nurses	Allied Health Professionals	Ancillary and Administrative Staff	*p* ^#^
US	7.1%	2.5%	6.7%	6.8%	13.3%	0.017
MRI	22.5%	5.0%	21.3%	22.3%	44.8%	<0.001
PET	26.3%	19.0%	22.7%	16.5%	49.5%	<0.001
CT	10.4%	0.0%	4.7%	9.7%	31.4%	<0.001

^#^ *p*-values were calculated using the chi-square test. US, ultrasound; MRI, magnetic resonance imaging; PET, positron emission tomography; CT, computerized tomography.

**Table 5:** Association between training and potential level of exposure to score by question level of difficulty. The adjusted coefficient, as calculated using linear regression, represents the difference among categories after controlling for the potential confounder.

Question Difficulty			Standard Score Mean (SD)	Adj. Coefficient (95% CI) *	*p* ^#^
All	Received training	No	−0.18 (1.00)	Ref.	
		Yes	0.42 (0.88)	0.16 (−0.01–0.32)	0.066
Low		No	−0.12 (0.97)	Ref.	
		Yes	0.27 (1.02)	0.06 (−0.12–0.23)	0.544
Moderate		No	−0.21 (0.98)	Ref.	
		Yes	0.50 (0.85)	0.28 (0.11–0.46)	0.002
High		No	−0.09 (0.99)	Ref.	
		Yes	0.20 (1.00)	0.03 (−0.17–0.24)	0.744
All	Potential exposure	No	−0.21 (0.99)	Ref.	
		Yes	0.19 (0.97)	0.16 (0.01–0.31)	0.036
Low		No	−0.14 (1.01)	Ref.	
		Yes	0.13 (0.98)	0.09 (−0.08–0.25)	0.291
Moderate		No	−0.23 (0.98)	Ref.	
		Yes	0.21 (0.97)	0.20 (0.04–0.36)	0.015
High		No	−0.11 (0.89)	Ref.	
		Yes	0.11 (1.08)	0.10 (−0.08–0.29)	0.266

* Adjusted to age, sex, seniority, employee type, and exposure/past training; ^#^ *p*-values were calculated using linear regression.

## Data Availability

The authors declare that they had full access to all of the data in this study and the authors take complete responsibility for the integrity of the data and the accuracy of the data analysis. Questionnaire data will be made available for reasonable requests.

## Supplementary Materials

The following supporting information can be downloaded at https://www.mdpi.com/article/10.3390/healthcare13080958/s1, Questionnaire data.

## Appendix A

**Table A1:** Distribution of employees by hospital wing.

Wing	Number of Participants	%	Exposure to Ionizing Radiation
Internal medicine	141	29.4%	57 High/84 Low
Surgical	164	34.2%	127 High/36 Low
Pediatrics	45	9.4%	37 High/8 Low
General emergency	25	5.2%	25 High/0 Low
Rehabilitation	41	8.6%	0 High/41 Low
Porter services	34	7.1%	0 High/34 Low
Nutritional services	29	6.1%	0 High/29 Low

- Internal medicine wing included internal medicine departments, cardiology department, and cardiac intensive care unit.
- Surgical wing included general surgery, operating room, orthopedics, oncological orthopedics, anesthesia, urology, neurosurgery, and recovery.
- Pediatric wing included pediatric emergency department, pediatric intensive care unit, and neonatal intensive care unit.

## Appendix B

**Table A2:** Comparison of mean (SD) scores among employees based on previous training.

Employee Type	Received Training	Difficulty Level			
		All	Low	Medium	High
Physicians	No	0.71 (0.81)	0.45 (0.66)	0.69 (0.80)	0.60 (1.31)
	Yes	0.98 (0.65)	0.72 (0.61)	0.95 (0.75)	0.60 (1.15)
	*p*-value	0.054	0.021	0.070	0.993
Nurses	No	0.04 (0.76)	0.18 (0.08)	−0.14 (0.80)	−0.03 (0.95)
	Yes	0.19 (0.67)	0.29 (0.85)	0.10 (0.80)	−0.07 (0.80)
	*p*-value	0.253	0.433	0.089	0.772
Allied health professionals	No	−0.10 (0.68)	0.11 (0.69)	−0.22 (0.89)	−0.25 (0.79)
	Yes	0.01 (0.83)	−0.22 (1.21)	0.37 (0.79)	−0.04 (0.72)
	*p*-value	0.539	0.195	0.003	0.225
Ancillary and administrative staff	No	−1.07 (0.86)	−1.00 (0.94)	−0.88 (0.85)	−0.46 (0.64)
	Yes	−0.64 (1.01)	−1.06 (1.36)	0.15 (0.67)	−0.23 (0.77)
	*p*-value	0.120	0.836	<0.001	0.276

## Appendix C

**Table A3:** Comparison of scores among employees who worked in high potential exposure departments and those who did not.

Employee Type	Potential Exposure	Difficulty Level			
		All	Low	Medium	High
Physicians	Low	0.77 (0.75)	0.46 (0.66)	0.75 (0.72)	0.68 (1.25)
	High	0.89 (0.74)	0.66 (0.63)	0.85 (0.83)	0.55 (1.22)
	*p*-value	0.385	0.094	0.500	0.595
Nurses	Low	−0.10 (0.76)	0.11 (0.89)	−0.32 (0.82)	−0.12 (0.80)
	High	0.18 (0.71)	0.26 (0.77)	0.06 (0.77)	0.00 (0.95)
	*p*-value	0.029	0.272	0.006	0.440
Allied health professionals	Low	−0.22 (0.65)	0.02 (0.76)	−0.33 (0.86)	−0.33 (0.55)
	High	0.23 (0.78)	0.05 (1.03)	0.46 (0.72)	0.08 (1.07)
	*p*-value	0.003	0.888	<0.001	0.047
Ancillary and administrative staff	Low	−1.03 (0.90)	−0.98 (1.04)	−0.79 (0.85)	−0.47 (0.5)
	High	−1.03 (0.85)	−1.05 (0.91)	−0.74 (0.96)	−0.40 (0.85)
	*p*-value	0.997	0.714	0.769	0.592
