# Supplementary material for: Knowledge and Awareness of Ionizing Radiation Harms Among Hospital Employees at a Large Tertiary Medical Center: Findings from a First-of-Its-Kind Study in Israel
Source: Healthcare (Basel). 2025 Apr 21;13(8):958. doi: 10.3390/healthcare13080958 (PMC12026994; doi:10.3390/healthcare13080958)
Supplement: Supplementary file 1 [file healthcare-13-00958-s001.zip › healthcare-3438225-supplementary.pdf]

Dear Employee ,

I am Ghassan Makhoul, an X-ray technician and graduate student at the Tel-Aviv University School of Public Health.

For research I'm conducting for my thesis paper, advised by Professor Gil Fire and Dr. Tomer Ziv-Baran, I ask you to answer a questionnaire that examines the level of knowledge and awareness of ionizing radiation damage.

The questionnaire is anonymous and will be exclusively used for research purposes.

Thank you in advance,

Ghassan

Part 1 (Demographic characteristics)

1) Age: ______
2) Gender:
    1) Male
    2) Female
3) Department: _____________
4) Occupation:
    1) Doctor
    2) Nurse
    3) Intern
    4) Medical assistant
    5) Secretary
    6) Personal support worker/orderly
    7) Housekeeping staff
    8) Dietitian
    9) Physiotherapist
    10) Psychologist

5) Year of receipt of professional license/the beginning of work in profession: _________

Part 2 (Professional characteristics related to ionizing radiation)

6) Did you receive special training regarding ionizing radiation via medical simulation or attend a lecture/training in which the harms of ionizing radiation were explained to you in an organized fashion?
    1. Yes
    2. No
7) If your answer to the previous question (6) is yes, where did you get the training/explanation? (It is possible to select more than one answer).
    1) As part of my studies ____ 2) At the medical center ____ 3) Other: ____
8) According to your own self-assessment, what is your level of knowledge and awareness about ionizing radiation damage (1-very low, 10- excellent)

    1 2 3 4 5 6 7 8 9 10

9) In your opinion, is it necessary to train employees and provide an explanation of the harms of ionizing radiation within the medical center?
    1) Yes
    2) No

10) On average, how many patients per week do you refer to the following tests?
   1) Not relevant to the profession.
   2) Computed Tomography (CT)
   3) X-ray imaging
   4) Ultrasound
   5) Magnetic Resonance (MRI)

11) Do previous tests done on the patient using methods that use ionizing radiation affect your decision whether to refer them for further tests with the use of ionizing radiation?
   1) Yes, frequently
   2) Yes, rarely
   3) No
   4) Not relevant to the profession

12) Do you <u>accompany</u> patients to imaging tests that are conducted using ionizing radiation?
   1) Yes, frequently
   2) Yes, rarely
   3) No
   4) Not relevant to the profession.

13) If you refer or accompany patients to imaging tests performed using ionizing radiation, do you explain to the patient or their companions about the test and the potential harms caused by it?
   1) Yes, frequently
   2) Yes, rarely
   3) No
   4) Not relevant to the profession

14) Are you present in the department when X-ray imaging is conducted (mobile unit)?
   1) Yes, always
   2) Yes, sometimes
   3) No
   4) Not relevant to the profession

Part 3 (Knowledge and awareness)

15) Of the following imaging methods, which is performed using ionizing radiation?
   A. Ultrasound Yes/No
   B. Computed Tomography (CT) Yes/No
   C. Magnetic Resonance (MRI) Yes/No
   D. Positron Emission Tomography (PET) Yes/No

16) Of the following tests, which are safe for a pregnant woman?
   A. Mammography
   B. Computed Tomography (CT)
   C. Chest X-ray with lead shield
   D. Ultrasound

17) Multiple brain CT scanning may cause:
A. Headache
B. Cataracts and turbidity of the lens of the eye
C. None of the above
D. I do not know

18) Of the following, which age group is the most sensitive to ionizing radiation?
A. Elderly
B. Adults
C. Children
D. All age groups are equally sensitive
E. I do not know.

19) Which gender is <u>more</u> sensitive to ionizing radiation?
A. Male
B. Female
C. Both genders equally
D. I do not know

20) Which of the following arguments is correct regarding a developing fetus?
A) The most significant effect of ionizing radiation on the fetus would be in the third trimester.
B) A fetus exposed to a low and acceptable level of radiation will have a higher risk of postpartum developmental delays.
C) Developing embryos are more sensitive to ionizing radiation than adult embryos because their cells are in the highest division phase.

21) Which of the following organs is known to be the most sensitive to ionizing radiation?
A. Thyroid
B. Lungs
C. Skin
D. Bones
E. Bone marrow
F. Brain
G. I do not know

22) What material is currently used to make protective clothing against ionizing radiation?
A. Aluminum
B. Lead
C. Iron
D. Plastic

23) What is the permitted annual dose of radiation per patient? (20 MSV/year is the annual dose permitted per employee)
A. 50 MSV
B. 20 MSV
C. 10 MSV
D. Unlimited
E. I do not know

24) In comparison to the radiation that a patient receives from one chest imaging, what is the radiation quantity for each of the following tests **in multiples of chest images (e.g. a pelvic CT equals X chest images)**

Test	I don't know	No ionizing radiation used	less than one chest image	equals one chest image	2-5 chest images	10-20 chest images	50-200 chest images	200-400 chest images	more than 400 chest images
Head CT									
Abdominal CT									
Chest CT									
Pelvic CT									
MRI									
Ultrasound									
Mammography									

25) For protection against ionizing radiation while taking a chest X-ray in the department (mobile unit), you need:

A) To stand behind a wall/pillar
B) To stand at least 1.8 meters away from the X-ray tube
C) No protection is needed
D) Leave the room

26) You meet with a patient who underwent a mapping a few minutes ago in order to reduce the dose of radiation you will receive:

A. I do not stand beside a patient who has undergone mapping without protective clothing against ionizing radiation.
B. Trying to shorten the meeting time.
C. I have already been exposed, and there is no need to shorten the meeting time.
D. There is no need to be protective at all from a patient who underwent a mapping a few minutes ago.

27) After you accompanied a patient to computed tomography (CT) scan of the whole body due to a fear of ionizing radiation damage:

1. Return the patient to the department immediately so as not to radiate the staff or other patients.
2. Protect yourself well before leaving the examination room and only then leave for the department.
3. No need for protection, a patient after a CT does not pose a danger in terms of ionizing radiation.

28) Once exposed (in test rooms or operating rooms):

1. I shield myself and use radiation measurement tags before entering the room.
2. I shield myself before entering the room but do not use radiation measurement tags.
3. I do not shield myself before entering a room if I'm entering for a short time.
4. I am already at an age where I do not have to be protected from ionizing radiation.
5. Not relevant to the profession.

Answer Correct/Incorrect to questions 29-32

29) When performing a computed tomography (CT) scan it is recommended to leave only one staff member in the examination room with protective clothing for supervision.
Right/Wrong

30) It is forbidden to perform an MRI examination more than 5 times a year on the same patient for fear of ionizing radiation damage.
Right/Wrong

31) A cardiac mapping test is not harmful in terms of ionizing radiation to those around the patient, rather only to the patient.
Right/Wrong

32) Abdominal CT scans are prohibited to be done more than 5 times a year for the same patient for fear of ionizing radiation damage.
Right/Wrong
